# The role of hypoxia-associated miRNAs in acquired sensorineural hearing loss

**DOI:** 10.3389/fncel.2022.916696

**Published:** 2022-08-05

**Authors:** Sina Safabakhsh, Printha Wijesinghe, Morgan Nunez, Desmond A. Nunez

**Affiliations:** ^1^Division of Otolaryngology—Head and Neck Surgery, Department of Surgery, Faculty of Medicine, The University of British Columbia, Vancouver, BC, Canada; ^2^Faculty of Medicine, University of Aberdeen, Aberdeen, United Kingdom; ^3^Division of Otolaryngology—Head and Neck Surgery, Gordon and Leslie Diamond Health Care Centre, Vancouver General Hospital, Vancouver, BC, Canada

**Keywords:** hearing loss, microRNAs, hypoxia, ischemia, oxidative stress, hypoxamiRs

## Abstract

**Introduction**: Sensorineural hearing loss (SNHL) is a prevalent sensory deficit presenting commonly as age-related hearing loss. Other forms of SNHL include noise-induced and sudden SNHL. Recent evidence has pointed to oxidative stress as a common pathogenic pathway in most subtypes of acquired SNHL. MicroRNAs (miRNAs) are small non-coding RNA sequences that suppress target mRNA expression and affect downstream processes. Many studies have shown that miRNAs are integral biomolecules in hypoxia-adaptive responses. They also promote apoptosis in response to oxidative stress resulting in SNHL. Our hypothesis is that miRNAs are involved in the pathophysiological responses to hypoxia and oxidative stress that result in SNHL. This study reviews the evidence for hypoxia-adaptive miRNAs (hypoxamiRs) in different types of acquired SNHL and focuses on miRNAs involved in hypoxia driven SNHL.

**Methods**: Electronic bibliographic databases PubMed, Ovid MEDLINE, Ovid EMBASE, and Web of Science Core Collection were searched independently by two investigators for articles published in English from the inception of individual databases to the end of July 2020. The text word or medical subject heading searches of all fields, titles, abstracts, or subject headings depending on the database were undertaken with combinations of the words “microRNAs”, “hypoxia”, “hypoxamiRs”, “oxidative stress”, “ischemia” and “hearing loss”. The reference lists of studies meeting the inclusion criteria were searched to identify additional relevant studies. The inclusion criteria included relevant clinical studies with human subjects, animals, and *in vitro* experiments. The risk of bias was assessed using the Cochrane risk of bias assessment tool for human studies and the Systematic Review Center for Laboratory animal Experimentation (SYRCLE) a risk of bias assessment tool for animal model and *in vitro* studies.

**Results**: A total of 15 primary articles were selected for full text screening after excluding duplicates, reviews, retracted articles, and articles not published in English. All nine articles meeting the study inclusion criteria were from animal or *in vitro* model studies and were assessed to be at low risk of bias. miRNAs miR-34a and miR-29b were reported to be involved in SNHL in inner ear cell models exposed to oxidative stress. Signaling pathways Sirtuin 1/peroxisome proliferator-activated receptor gamma coactivator-1-alpha (SIRT1/PGC-1α), SIRT1/p53, and SIRT1/hypoxia-inducible factor 1-alpha (HIF-1α) were identified as underlying pathways involved in acquired SNHL.

**Conclusion**: There is evidence that miR-34a and -29b are involved in hypoxia-driven and other causes of oxidative stress-related acquired SNHL. Further studies are required to determine if these findings are clinically applicable.

## Introduction

### Background

Hearing loss has been ranked as the fifth leading cause of years lived with disability, higher than other chronic diseases such as diabetes, dementia, and chronic obstructive pulmonary disease (Global Burden of Disease Study 2013 Collaborators, 2015). At the societal level, hearing loss has a significant economic impact and is estimated to cost over 750 billion USD globally (World Health Organization, 2017). Sensorineural hearing loss (SNHL) an increasingly prevalent sensory deficit estimated to affect 1.57 billion people, presents most commonly as age-related hearing loss (ARHL) which affects up to 62.1% of individuals 50 years and older worldwide (GBD 2019 Hearing Loss Collaborators, 2021).

The clinical presentation of SNHL can differ depending on the specific form of SNHL. However, hearing loss and tinnitus are ubiquitous symptoms, and in conditions which also affect the vestibular apparatus, vertigo can occur. ARHL is believed to be due to the accumulation of reactive oxygen species (ROS) within hair cells that eventually leads to cell death as part of normal aging (Riva et al., 2007). Aside from ARHL, other forms of acquired SNHL include sudden SNHL, and noise-induced SNHL. The mechanisms underlying sudden SNHL are not fully understood, however, the most accepted mechanism is vascular compromise which suggests that a sudden disruption of the circulation that supplies the cochlea leads to ischemia and associated hypoxic-cell death (Kim et al., 2016). Noise-induced SNHL usually occurs because of irreversible cumulative acoustic trauma to cochlear hair cells, leading to cell death.

### Acquired SNHL and hypoxia/oxidative stress-related miRNAs

The ear is comprised of an outer, middle, and inner component. The inner ear houses the main sensory organ responsible for the special sense of hearing. This sensory organ is called the organ of Corti and it is located within the cochlea. Damage to the sensory cells of the organ of Corti or of the vestibulocochlear nerve (cranial nerve 8) that carries signals to the brain, results in SNHL. The damage could be induced by direct trauma, vascular compromise leading to cellular hypoxia, or secondary to the ototoxic effects of various drugs. The sensitivity of the cochlear vasculature to circulating inflammatory factors places the ear at considerable risk of damage (Trune and Nguyen-Huynh, 2012). Tissue hypoxia secondary to ischemia results in oxidative stress directly through reduced aerobic metabolic mitochondrial electron transport chain activity partly due to inhibition of cytochrome oxidase (Raedschelders et al., 2012) and through mitochondrial dysfunction secondary to increased intra-mitochondrial calcium accumulation (Starkov et al., 2004). More oxygen electron transfer under these conditions occurs by multiple single electron transfer steps resulting in an increase in free oxygen radicals. Furthermore, reperfusion following ischemia leads to reactive oxygen species (ROS) generation overwhelming ROS degradation capacity (Raedschelders et al., 2012). Oxidative stress and high levels of ROS which occur in ARHL (Fujimoto and Yamasoba, 2014), sudden SNHL, and noise trauma induced SNHL suggest a common oxidative stress pathogenetic pathway in acquired SNHL. Oxidative stress is associated with ROS production which, in high concentrations, leads to cellular apoptosis and hearing loss. Despite the significant impact on individuals and society, effective treatments for SNHL beyond electro-mechanical devices namely hearing aids or cochlear implants are mostly lacking (Nunez et al., 2020). Changes in miRNA expression in response to hypoxia exert an effect on cells by regulating the function of genes involved in signaling pathways (Gupta et al., 2018). A link between miRNAs and oxidative stress is described in other ischemia associated conditions such as ischemic strokes, and giant cell arteritis (Croci et al., 2016; Devaux et al., 2016; Chen et al., 2017) with miRNAs being proposed as diagnostic or prognostic biomarkers.

miRNAs are endogenous, short regulatory RNA molecules, that modulate target gene expression at the post-translational level by guiding the RNA-induced silencing complex (RISC) to miRNA target sites in the 3’ untranslated region of mRNAs, leading to mRNA degradation or the inhibition of translation (Bartel, 2004). miRNAs are expressed in normal inner ear cells, and play an essential role in their development, differentiation, and survival (Friedman et al., 2009; Ushakov et al., 2013). Changes in miRNA expression have been, well documented in animal models of ARHL (Xiong et al., 2015; Xue et al., 2016) and shown to induce mitochondrial dysfunction and hair cell loss via downregulation of Sirtuin 1/ peroxisome proliferator-activated receptor-gamma coactivator 1α (SIRT1/PGC-1α) signaling in aged mice. SIRT1 regulates intracellular oxidative stress by the deacetylation of its substrates including PGC-1α, a transcriptional co-regulator that binds to numerous transcription factors to promote mitochondrial biogenesis and oxidative metabolism (Someya et al., 2010). Hence, it is reasonable to explore the evidence for changes in miRNAs associated with SNHL. Studies have documented the role of miRNAs in the cochlea in response to hypoxic stress. Following ROS production in the inner ear, changes in miRNAs alter mRNA expression; specifically, upregulation of miR-29a, miR-200c, and miR-17 occurs which in turn, downregulates insulin-like growth factor 1 (IGF-1), alters (IGF-1) mediated signaling and contributes to hearing loss (Ushakov et al., 2013).

Many studies have shown that specific miRNAs (hypoxamiRs) are integral biomolecules in hypoxia-adaptive responses (Gupta et al., 2018). Devaux et al. (2016) found that the level of circulating miR-124-3p after cardiac arrest and cardiomyocyte ischemia was associated with survival outcome in patients who experienced out-of-hospital cardiac arrest. A number of hypoxamiRs have been proposed as diagnostic or prognostic markers namelymiR-223 in stroke (Chen et al., 2017), let-7e, miR-15b, -16, -20b, -25, -26b, -27b, -28–5p, -126, -195, -335, and -363 in peripheral arterial disease (Stather et al., 2013), and miR-342, -191 and -510 in diabetes mellitus (Hezova et al., 2010).

### Objective

Therefore, this review aims to identify SNHL related hypoxia-associated miRNAs (hypoxamiRs) with a view to facilitating the development of new SNHL diagnostic, prognostic and therapeutic tools.

## Methods

### Electronic search strategy

The electronic bibliographic databases PubMed, Ovid MEDLINE, Ovid EMBASE, and Web of Science Core Collection were searched for articles published in English from the inception of the database to the end of July 2020. A text word or MeSH terms search of all fields, titles, abstracts, or subject headings depending on the database was undertaken with combinations of the words “microRNAs”, “hypoxia”, “hypoxamiRs”, “oxidative stress”, “ischemia” and “hearing loss”. The reference lists of studies meeting the inclusion criteria were further searched to identify additional relevant studies.

### Article selection

Two investigators (SS and MN) independently screened the titles and abstracts yielded by the search to select full text articles that matched the purpose of this review. Primary articles investigating ARHL, sudden SNHL, noise-, chemical-, ototoxic drug- or neurotoxic drug-induced hearing loss, or other forms of acquired SNHL such as: published clinical studies, case series, and case reports with human participants including adults, children, or neonates who had documented SNHL based on otoacoustic emission, brain stem evoked audiometry, or masked bone conduction pure tone audiometry tests were included. Additionally, published experimental studies on mammalian animal models and in *in vitro* cell models were included. All types of review articles, non-full text articles, books, conference abstracts, letters, surveys, articles not published in the English language, studies lacking *a priori* experimental design, and primary studies on non-mammals (example: avian, amphibians) were excluded.

Human participant studies where exposure to hypoxia or ischemia/oxidative stress was documented by objective measures, such as altered plasma glutathione, thioredoxin or clinical measures such as the APGAR score and studies including patients diagnosed with clinical conditions affecting oxygen saturation levels were targeted for inclusion. Likewise, animal model and *in vitro* cell studies, where hypoxia or tissue ischemia was evidenced by production of ROS or expression of hypoxia-associated genes (e.g., HIF-1α, CDKN1B), and or induced by physiological or pharmacological approaches were included. Human studies without evidence of exposure to hypoxia or tissue ischemia that is oxygen saturation levels of 95%–100% and better, and those studying conditions without an ischemia-driven pathophysiology were excluded. Animal model and *in vitro* cell model studies carried out under normoxic conditions or where there was no ROS production or expression of hypoxia-associated genes were also excluded.

Any disagreements on the inclusion of individual studies were resolved by the majority opinion in consultation with the senior investigator (DN).

### Data extraction

Two investigators (SS, MN) extracted data from the included studies using standard data extraction forms. For the human participants, animal model, and *in vitro* cell model studies: publication year, authors, study design, type of SNHL investigated, sample size, mean ages of treatment and control group subjects, methods of hypoxia induction, reagents used to induce hypoxia, dosage, exposure time, samples targeted for miRNA extraction, techniques used to determine miRNA expression levels, validation methods, reported differentially expressed miRNAs, confirmed or predicted target genes, and gene enrichment or functional enrichment pathway analysis findings were recorded.

Study investigators undertook data extraction independently. Discrepancies and inadequacies in the data extracted by individual investigators were resolved in consultation with the senior investigators (PW or DN). If relevant data was not reported in an article, the article’s corresponding author was contacted twice to obtain the data.

### Outcomes

The primary outcome was the identification of microRNAs involved in different types of acquired SNHL in response to hypoxic, oxidative stress, or ischemic insults (hypoxamiRs). A hypoxamiR was defined for this review as a miRNA that was differentially expressed based on qPCR when compared with controls at a statistically significant (*p* < 0.05) level, or with a minimum 2-fold intergroup difference; or *via* other validated methods such as Western blot, reporter assay, sequencing; or similar objective methods. The secondary outcomes were the predicted and validated target gene(s), target genes enriched or functionally enriched pathways, and the suggested underlying molecular targets or pathological actions of the hypoxamiRs. HypoxamiR target genes were similarly defined.

### Assessment of study quality and risk of bias

The quality of evidence and risk of bias were assessed independently by three investigators (SS, MN, and DN). The Systematic Review Center for Laboratory animal Experimentation (SYRCLE; Hooijmans et al., 2014) tool based on the Cochrane risk of bias tool adjusted for aspects of bias that play a specific role in animal intervention studies was used for animal studies. It was also used for *in vitro* cell model studies, as there is currently no widely accepted standardized risk of bias tool for *in vitro* studies. The SYRCLE tool assesses selection bias (related to sequence generation, baseline characteristics, allocation concealment), performance bias, detection bias, attrition bias, reporting bias, and other sources of bias. The overall score of high, low, or indeterminate based on the SYRCLE risk of bias tool was decided by majority opinion. A high-quality study was defined as one with a low risk of bias in all the assessed domains.

### Data presentation

A narrative synthesis was planned. The Cochrane Collaboration’s Review Manager version 5.3 was used to generate Preferred Reporting Items for Systematic Reviews and Meta-Analysis (PRISMA) flow diagram of the results of the electronic searches and to visualize the risk of bias assessments (Mcguinness and Higgins, 2021).

## Results

**Figure 1:**
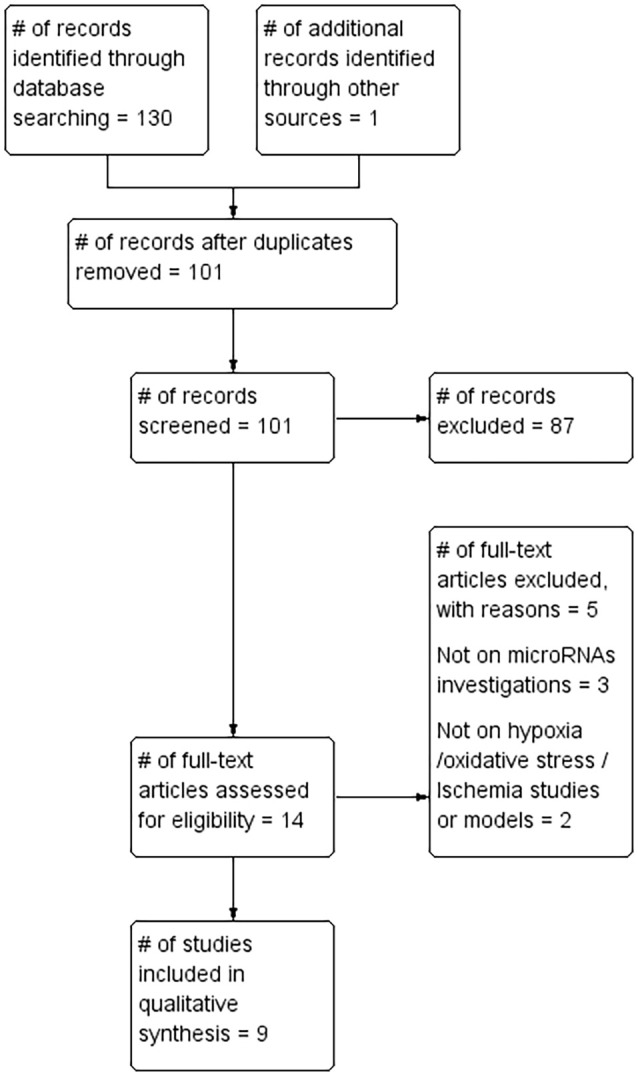
Preferred Reporting Items for Systematic Reviews and Meta-Analyses (PRISMA) flow chart.

A total of 130 records were identified by database searches. One additional article was found by searching the reference list of included articles. A total of 101 article abstracts were screened after duplicates were removed. A total of 87 that failed to meet study inclusion criteria were excluded. The full text of the remaining 14 articles was reviewed and a further five were excluded for failing to meet the inclusion criteria. The remaining nine articles (Tables s 1, 2) formed the basis of this review (Figure 1).

**Table 1:** Characteristics of the included *in vitro* cell models/studies.

**Author and Year**	**Wang et al. (2010)**	**Kim et al. (2014)**	**Xiong et al. (2015)**	**Xue et al. (2016)**	**Lin et al. (2017)**	**Hao et al. (2019)**	**Xiong et al. (2019)**	**Lai et al. (2020)**
**Cell lines used**	HEI-OC1 (Permissive - 33°C 10% CO_2_)	HEI-OC1 (Permissive - 33°C 5% CO_2_)	HEI-OC1 (Permissive - 33°C 10% CO_2_)	HEI-OC1 (Permissive - 33°C 7% CO_2_)	HEI-OC1 (Permissive - 33°C 10% CO_2_)	SK-N-MC and SH-SY5Y cells (37°C 5% CO_2_)	HEI-OC1 (Permissive - 33°C 10% CO_2_)	HEI-OC1 (37 °C 5% CO_2_)
**Type of SNHL investigated**	Ototoxic drug-induced	Ototoxic drug-induced	ARHL	ARHL	Diabetes-related hearing loss	ARHL	ARHL	Ototoxic drug-induced
**Study design—Experimental**								
Control group/s	No treatment/s	No treatment/s	No treatment/s	No treatment/s	Normal glucose (5.5 mmol)	PBS group	No treatment/s	No treatment/s
Treatment group/s	t-BHP	-Cisplatin -Cisplatin and β-Lap (at different concentrations)-Cisplatin (+/–) and DHIQ -2 siRNA transfection groups (pre-miR-34a and control)-Cisplatin (+/–) and 4 transfection groups (NQO1, PARP-1, p53 and control)-Cisplatin and β-Lap (+/–) and 4 transfection groups (NQO1, PARP-1, p53 and control)	-4 transfection groups at different concentrations (miR-34a mimic, mimic control, miR-34a inhibitor, inhibitor control)-Resveratrol (0, 2, 5, 10 μM) and 2 transfection (miR-34a mimic and mimic control) groups -H_2_O_2_ (+/–) and resveratrol (0, 5, 10 μM) and 2 transfection (miR-34a mimic and mimic control) groups-Resveratrol (+/–) and 2 transfection (miR-34a mimic and mimic control) groups, -resveratrol alone,-5-FU alone and -recevatol + 5-FU group	-H_2_O_2_-H_2_O_2_ + 5 transfection groups (miR-29b mimic, mimic control, miR-29b inhibitor, plk0.1-SIRT1 and plk0.1-scrample)	-High glucose -High glucose + 8 transfection groups(miR-34a mimic, mimic control; miR-34a-inhibitor, inhibitor control; si-SIRT1 and a negative control; si-HIF-1αand a negative control)-High glucose + 2 transfection groups (pECE-Flag-SIRT1 and si-MOCK/negative control)	-H_2_O_2_ + negative control- H_2_O_2_ + transfected with MIAT-H_2_O_2_ + transfected with anti-miR-29b	- H_2_O_2_-UCDA-UCDA+ H_2_O_2_- H_2_O_2_ + SRT- H_2_O_2_ + 4 transfection groups (Parkin, PGC-1α, Drp1 and control/ scrambled siRNA)	-Gentamicin-Gentamicin +1 transfection group (IESC-ex) -Gentamicin +3 transfection groups (IESC-ex, IESC-miR-182-5p-ex and IESC-miR-NC-ex)
Dosage(s) (hypoxia inducing agent)	0, 50, 100, 200 μM	20 μM	50 μM	50 μM	30 mmol	No data	1 mM	2 mM
Exposure time	12 h	up to 24 h	1 h	1 h	48 h	not disclosed	1 h	not disclosed
Harvesting interval(s)	immediately	0, 6, 12, 18, 24 h	48, 72 h	72 h	48 h	not disclosed	6, 12, 24 h	not disclosed
Techniques used to confirm hypoxia-induction/ROS production	-DCFH-DA assay (Flow cytometric analysis)-Cell viability assay (CCK-8)-Annexin-V FITC, Apoptosis Kit (Flow cytometric analysis)	-Cell proliferation assay (MTT) -Chemiluminescent, PARP Assay Kit -Fluorescent SIRT1 Assay Kit	-Immunocyto-chemistry-Cell viability assay (MTS)-Annexin-V FITC, Apoptosis Kit (Flow cytometric analysis)	- Cell proliferation assay (MTT)-Annexin V-FITC Apoptosis Detection Kit (Flow cytometric analysis)-JC-1 Fluorescence Kit	-Cell viability assay (MTT)-Annexin V-FITC Apoptosis Detection Kit (Flow cytometric analysis)	-Cell proliferation assay (MTT)-Annexin-V FITC, Apoptosis Kit (Flow cytometric analysis)	-Cell viability assay (MTS)-Immunocyto-chemistry (MitoSOX)-JC-1 Florescence Kit (Flow cytometric analysis)-ATP Assay -Western blotting and densitometry - qRT-PCR	-Superoxide dismutase assay kit -Catalase assay kit -Glutathione peroxidase activity -LDH Cytotoxicity Assay Detection Kit
**MicroRNA investigations**								
Samples used to extract RNAs	cell pellets	cell pellets	cell pellets	cell pellets	cell pellets	cell pellets	cell pellets	cell pellets
Technique used to determine the gene expression	MiRNA microarray	qRT-PCR	qRT-PCR	qRT-PCR	qRT-PCR	qRT-PCR	qRT-PCR	-qRT-PCR
Validation methods	qRT-PCR	Western blotting and densitometry	No data	No data	No data	No data	No data	No data
Reported differentially expressed miRNAs / candidate miRNAs	-miR-133a*/-17/-191*/-214/-467b*/-690/-691/ and miR-122/-27b*/-28*/-335–5p/-377/-383/-675–3p/-743b-5p/-871/-874-miR-29a/-203	miR-34a	miR-34a	miR-29b	miR-34a	miR-29b	miR-34a	miR-182–5p
**Gene expression studies**								
Technique used to determine the gene expression	-mRNA Microarray -TargetScan version 5.1	No data	qRT-PCR	qRT-PCR	qRT-PCR	qRT-PCR	qRT-PCR	qRT-PCR
and Validation methods	qRT-PCR	Western blotting and densitometry	-Western blotting and densitometry-Luciferase assay-Immunocyto-chemistry	Western blotting and densitometry	Western blotting and densitometry	-Western blot, -Luciferase assay	-Western blotting and densitometry -Immunocyto-chemistry	Western blotting and densitometry
Reported predicted and/or validated gene(s)	-CCND2, ATF7IP-IGF-1, PIK3R1, PTPN11-FOS, SOS2, PPP3R1, PPM1B, FLNC, PPP5C	SIRT1, PARP-1, p53, NF-kB p65	SIRT1, p53, acetyl p53, acetyl-FOXO1, 4HNE	SIRT1, PGC-1α	SIRT1, HIF-1α	MIAT, SIRT1, PGC-1α	SIRT1, PINK1, Parkin, PGC-1α, TFAM, COX4, SOD1, TOmm20, 4HNE, LAMP1	FOXO3, Bcl-2, Bax
Reported gene enrichment or functional enrichment pathway	IGF-1 signaling, MAPK signaling	miR-34a-p53-SIRT1-PARP-1	p53 - mir-34a-SIRT1	miR-29b-SIRT1-PGC-1α	miR-34a- SIRT1/HIF-1α	MIAT-miR-29b-SIRT1-PGC-1α	miR-34a-SIRT1	miR-182–5p-FOXO3-Bax-Bcl2

Study characteristics of eight primary investigations that fulfilled the study inclusion criteria are summarized. ARHL, age related hearing loss; HEI-OC1, House Ear Institute-Organ of Corti 1; IESC, inner ear stem cells; IESC-ex, exosomes derived from IESCs; NC, negative control; qRT-PCR, quantitative reverse transcription polymerase chain reaction; ROS, reactive oxygen species. NQO1, NADH:quinone oxidoreductase 1; SIRT1, sirtuin 1; PGC-1α, peroxisome proliferator-activated receptor gamma coactivator 1-alpha; HIF-1α, *hypoxia-inducible factor 1*-alpha; PINK1, PTEN induced kinase 1; PARP-1, poly (ADP-ribose) polymerase 1; MIAT, myocardial infarction associated transcript; 4HNE, 4-hydroxy-2-nonenal; TFAM, mitochondrial transcription factor A; TOmm20, translocase of outer mitochondrial membrane 20; COX4, cytochrome c oxidase subunit 4; SOD1, superoxide dismutase; NF-kB, nuclear factor kappa B; LAMP1, lysosomal-associated membrane protein 1; FOXO1/3, forkhead box protein O 1/3; CCND2, cyclin D2; ATF71P, activating transcription factor 7-interacting protein 1; IGF-1, Insulin-like growth factor 1; PIK3R1, phosphoinositide-3-kinase regulatory subunit 1; PTPN11, tyrosine-protein phosphatase non-receptor type 11; FOS, proto-oncogene, AP-1 transcription factor subunit; SOS2, Ras/Rho guanine nucleotide exchange factor 2; PPP3R1, protein phosphatase 2B regulatory subunit 1; PPM1B, protein phosphatase 1B; FLNC, filamin-C; PPP5C, protein phosphatase 5 catalytic subunit; Bcl2, B-cell lymphoma 2; MAPK, mitogen-activated protein kinase. t-BHP, tertbutyl hydroperoxide, β-Lap, β-lapachone; UCDA, ursodeoxycholic acid; 5-FU, 5-fluorouracil; CCK-8, cell counting kit-8; DCFH-DA, 6-carboxy-2’,7’-dichlorodihydrofluorescein diacetate; FITC, fluorescein isothiocyanate; JC- 1,5,5’,6,6’-tetrachloro-1,1’,3,3’-tetramethyl-benzimidazolylcarbocyanine iodide (mitochondrial dye); MTT, 3-(4,5-dimethyl-2-thiazolyl)-2,5-diphenyltetrazolium bromide; MTS, 3-(4,5-dimethylthiazol-2-yl)-5-(3-carbo xymethoxyphenyl)-2-(4-sulfophenyl)-2H-tetrazolium; LDH, Lactate dehydrogenase; ATP, Adenosine triphosphate; *, Some miRNA hairpin precursors give rise to two excised miRNAs, one from each arm. An asterisk has been used to denote the less predominant form by Wang et al. (2010).

The nine studies included consisted of, two *in vitro* studies, one animal study, and six that included findings from both *in vitro* cell and animal studies. Four investigated ototoxic drug-induced SNHL, four investigated age-related hearing loss, and one investigated diabetes-related hearing loss. The study characteristics of the included *in vitro* cell model studies are summarized in Table 1 and the animal model studies in Table 2.

**Table 2:** Characteristics of included animal models/studies.

**Author and Year**	**Kim et al. (2014)**	**Xiong et al. (2015)**	**Kim et al. (2016)**	**Xue et al. (2016)**	**Lin et al. (2017)**	**Hao et al. (2019)**	**Xiong et al. (2019)**
**Animal (species)**	-C57BL/6 mouse -NQO1 knockout mice on C57BL/6 background	C57BL/6 mouse	-C57BL/6 mouse -NQO1 knockout mice on C57BL/6 background	C57BL/6 mouse	db mice	C57BL/6 mouse	-C57BL/6 mice -miR-34a +/– mice and SIRT1 transgenic mice on C57BL/6 background
**Type of SNHL investigated**	Ototoxic drug-induced	ARHL	Ototoxic drug-induced	ARHL	Diabetes-related hearing loss	ARHL	ARHL
**Study design—Experimental**							
Treatment group(s)	-Cisplatin-β-Lap + cisplatin-β-Lap alone	-Old-15 additional mice (2–3 months) for resveratrol treatment group (400 mg/kg/day for a period of 2 months)	-Cisplatin-Dunnione + cisplatin-Dunnione alone	Old	db/db mice (BKS.Cg-m+/+ Leprdb/J)	Old	-Old-Old + 3 treatment groups (at the age of 2 months, mice were started supplementary diet for 10 months with -high dose resveratrol (300 mg/kg/day) -low dose resveratrol (7.5 mg/kg/day) -rapamycinm (14 mg/kg/day)
Sample size	5 each	50	5 each	30	21	No data	varied
Age	8 weeks	12–16 months	8 weeks	12 months	8 weeks	12–16 months	12 months
Control group(s)	No treatment/s	-Young -13 additional mice (2–3 months) for resveratrol control group (standard chow)	No treatment/s	Young	db/m (C57BL/KSJ db/+)	Young	Young
Sample size	5	47	5	30	21	No data	varied
Age	8 weeks	1–2 months	8 weeks	1–2 months	8 weeks	1–2 months	2 months
**Induction of hypoxia/oxidative stress/ischemia**							
Method of induction	Chemical	Ageing	Chemical	Ageing	No data	Ageing	Ageing
Reagents used	Cisplatin		Cisplatin				
Dosage	16 mg/Kg		20 mg/Kg				
Exposure time	single intraperitoneal dose		single intraperitoneal dose				
Post-treatment interval	4 days		4 days				
Techniques used to confirm hypoxia-induction/ROS production	-H2-DCFDA Florescent Assay Kit -Chemiluminescent, PARP Assay Kit -Fluorescent SIRT1 Assay Kit -Fluorescent NAD+/NADH Detection Kit -Western blotting and densitometry (H2AX)-TUNEL Assay Kit	TUNEL Assay Kit	-H2-DCFDA Florescent Assay Kit -Chemiluminescent, PARP Assay Kit -Fluorescent SIRT1 Assay Kit -Western blotting and densitometry (H2AX)	-TUNEL Assay Kit-JC-1 Fluorescence Kit	TUNEL Assay Kit	-JC-1 Fluorescence Kit -TUNEL Assay Kit	-Immunocyto-chemistry -Western blotting and densitometry
**MicroRNA investigations**							
Samples used to extract RNAs	cochlea	cochlea	cochlea	cochlea	cochlea	cochlea	cochlea
Technique used to determine the gene expression	qRT-PCR	qRT-PCR	qRT-PCR	qRT-PCR	qRT-PCR	qRT-PCR	qRT-PCR
Reported differentially expressed miRNAs / candidate miRNAs	miR-34a	miR-34a	miR-34a	miR-29b	miR-34a	miR-29b	miR-34a
**Target gene expressions**							
Technique used to determine the gene expression	No data	qRT-PCR	qRT-PCR	qRT-PCR	qRT-PCR	qRT-PCR	No data
and Validation methods	-Western blotting and densitometry-Immunohisto-chemistry	-Immunohisto-chemistry, -Western blotting and densitometry	-Western blotting and densitometry-Immunohisto-chemistry	Western blotting and densitometry	Western blotting and densitometry	Western blot	-Immunocyto-chemistry, -Western blotting and densitometry, -Genotyping
Reported predicted and/or validated gene(s)	SIRT1, PARP-1, p53, NF-kB p65	SIRT1, p53, acetyl p53	SIRT1, PARP-1, p53, NF-kB p65, TNF-α	SIRT1, PGC-1α	SIRT1, HIF-1α	MIAT, SIRT1, PGC-1α	SIRT1, PINK1, Parkin, PGC-1α, NRF1, NRF2, TFAM, TOmm20, COX4, 4HNE, SOD1, p62, LC3-II
Reported gene enrichment or functional enrichment	miR-34a-p53-SIRT1-PARP-1	p53- miR-34a—SIRT1	miR-34a-p53-SIRT1-PARP-1	miR-29b-SIRT1-PGC-1α	miR-34a-SIRT1- HIF 1α	MIAT-miR-29b-SIRT1-PGC-1α	miR-34a-SIRT1

Study characteristics of seven primary articles that fulfilled the study inclusion criteria are summarized. ARHL, age related hearing loss; qRT-PCR, quantitative reverse transcription polymerase chain reaction; NQO1, NADH:quinone oxidoreductase 1; NAD+/NADH, nicotinamide adenine dinucleotide +/hydrogen; H2AX, histone H2A variant; SIRT1, sirtuin 1; PGC-1α, peroxisome proliferator-activated receptor gamma coactivator 1-alpha; HIF-1α, hypoxia-inducible factor 1-alpha; PINK1, PTEN induced kinase 1; PARP-1, poly (ADP-ribose) polymerase 1; MIAT, myocardial infarction associated transcript; NRF1/2, nuclear respiratory factor 1/2; TFAM, mitochondrial transcription factor A; TNF-α, tumor necrosis factor-*α*; TOmm20, translocase of outer mitochondrial membrane 20; COX4, cytochrome c oxidase subunit 4; 4HNE, 4-hydroxy-2-nonenal; SOD1, superoxide dismutase; NF-kB, nuclear factor kappa B; LC3-II, microtubule-associated protein light chain 3. β-Lap, β-lapachone; TUNEL, terminal deoxynucleotidyl transferase dUTP nick end labeling; JC- 1,5,5’,6,6’-tetrachloro-1,1’,3,3’-tetramethyl-benzimidazolylcarbocyanine iodide (mitochondrial dye); H2-DCFDA,2’,7’dichlorodihydrofluorescein diacetate.

**Figure 2:**
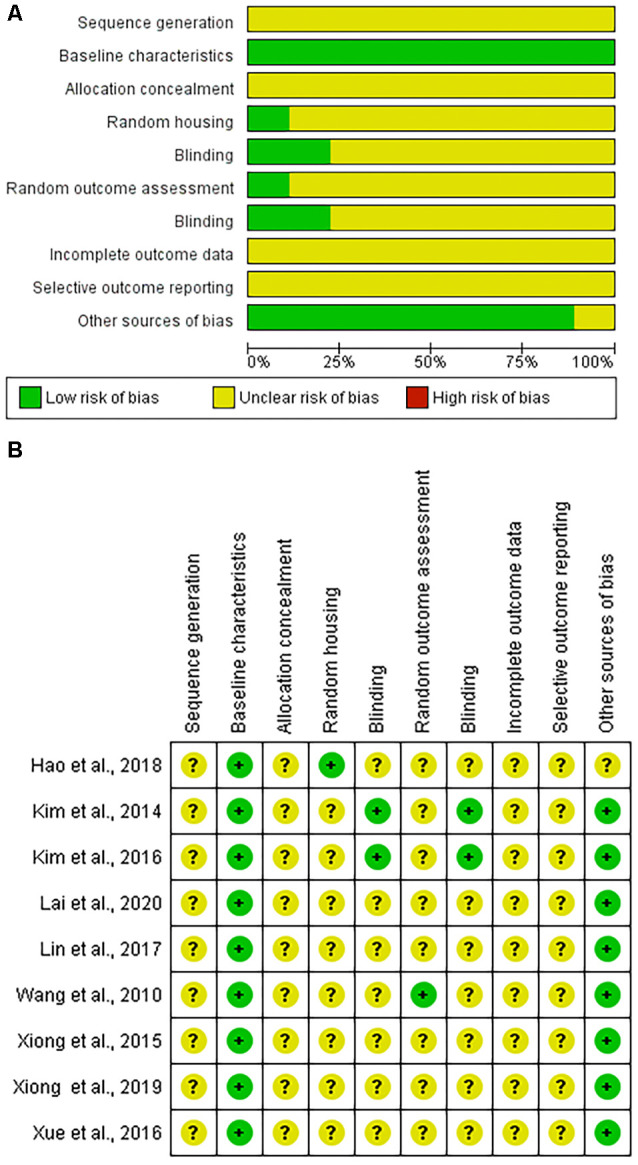
Systematic Review Center for Laboratory animal Experimentation (SYRCLE) risk of bias assessment. **(A)** Graphic representation summarizing the assessment of the nine included articles. **(B)** Summary of individual article assessments by SYRCLE domain.

The risk of bias was found to be unclear due to insufficient information or low in all articles using the SYRCLE tool. No article had areas that were deemed to be at a high risk of bias (Figure 2).

## Discussion

### The mediating role of miRNAs and ROS during oxidative stress

States of cellular stress, such as hypoxia or oxidative stress, are associated with increased levels of ROS (Raedschelders et al., 2012; Lushchak, 2014). Temporary and milder ROS release may lead to adaptive mechanisms facilitating cellular survival (Lushchak, 2014). However, prolonged ROS over-production and accumulation, such as in drug-induced ototoxicity or aging (Riva et al., 2007), can trigger maladaptive processes such as apoptosis or autophagy that lead to cellular damage and death.

There is now accumulating evidence to suggest that miRNAs play a role in mediating the signaling between excessive ROS production and the deleterious effects on cellular health and survival (Subramanian and Steer, 2010). Therefore, Wang et al. (2010) investigated the miRNA profile of the organ of corti cell damage because of oxidative stress by using increasing concentrations of the potent oxidant t-BHP to induce the release of ROS in HEI-OC1 cells. Following this treatment, the degree of ROS production and cell viability were correlated with miRNA and mRNA expression levels. They found that the expression levels of 35 miRNAs were upregulated and 40 downregulated while the transcription of 2,686 mRNAs were altered. Network analysis revealed miRNA changes consistent with activation of the pro-apoptotic MAPK and JNK signaling pathway (Wang et al., 2003) and inhibition of neuronal protection by downregulation of IGF-1 signaling. Specifically, MAPK signaling pathway miRNAs miR-383, -208a, -377, -181d, -377, and -874 and IGF-1 signaling pathway miRNAs miR-29a, -17, and -200c were responsible for oxidative stress induced cochlea cell death. The authors propose that drugs targeting the MAPK/JNK pathway may have anti-apoptotic effects of relevance to the treatment of SNHL.

### Cisplatin ototoxicity and hypoxic stress mediated hair cell damage

Cisplatin is a chemotherapeutic agent used to treat solid tumors (Cohen and Lippard, 2001). It has been associated with progressive hearing loss. The mechanism through which this occurs is not completely understood.

The study by Kim et al. (2014) demonstrated that the ototoxic mechanisms of cisplatin involve a p53 mediated upregulation of poly (ADP-ribose) transferase (PARP)-1 and miR-34a. This leads to a decrease in Nicotinamide adenine dinucleotide (NAD+) levels resulting in decreased Sirtuin 1 (SIRT1). A combination of mouse models and *in vitro* HEI-OC1 cell lines in this study showed that cisplatin induced oxidative stress that damaged hair cell DNA and hyperactivated PARP-1. PARP-1 activation at modest levels is important for DNA repair, however, excess PARP-1 activity leads to depletion of the co-factor NAD+ (Goodwin et al., 1978). Normally, SIRT1 would consume NAD+ to mediate a range of processes, including DNA repair. However, depleted NAD+ led to reduced SIRT1 activity and DNA repair mechanisms. Moreover, while it had been previously shown that cisplatin treatment led to inhibition of p53 expression that resulted in cytotoxicity, further downstream effects of p53 inhibition were not fully known. This study identified that reduced p53 levels led to overexpression of miR-34a. The increased miRNA-34a then suppressed SIRT1 translation. The reduced SIRT1 activity due to depleted NAD+ pool and translational block by miR-34a led to impaired DNA repair mechanisms resulting in hair cell damage and ototoxicity. Furthermore, Kim et al. (2014) obtained evidence that p53 silencing RNA transfection of HEI-OC1 cells reduced miR-34a overexpression and restored SIRT1 expression in cisplatin treated cells. However, no miR-34a silencing experiments were undertaken so there was no direct evidence that SIRT1 expression is controlled by miR-34a, though it is strongly implied.

### Potential novel ototoxicity treatments targeting oxidative stress

Kin et al.’s initial and follow-on studies identified that recovery of the available NAD+ pool by administration of Dunnione an antifungal and anti-tumor drug that is a substrate of antioxidant flavoprotein NADH: quinone oxidoreductase 1 (NQO1) ameliorated the ototoxic effects of cisplatin (Kim et al., 2014, 2016). Gentamicin ototoxicity associated oxidative stress was demonstrated in HEI-OC1 cells cultured under non-permissive conditions by an increase in Malondialdehyde (MDA) and a reduction in superoxide dismutase (SOD), both markers of oxidative stress (Ho et al., 2013). The addition of mouse inner ear stem cell derived exosomes to gentamicin treated HEI-OC1 cell cultures significantly reversed changes in MDA and SOD levels (Lai et al., 2020). These changes were accompanied by increased inner ear cell survival. Lai et al. (2020) studied the effect of exosomes derived from mouse inner ear stem cells (IESC-ex) on gentamicin toxicity in HEI-OC1 cells for evidence that miR-182-5p/FOXO3 was the pathway through which the exosomes were exerting their effect. They added increasing concentrations of IESC-ex to gentamicin-treated non-permissive cultured HEI-OC1 cells and identified a dose-dependant: reduction in gentamicin-induced oxidative stress, downregulation of miR-182-5p, upregulation of FOXO3 mRNA, and protein expression. It was further determined that exosomes derived from miR-182-5p transfected IESCs exacerbated these effects compared with exosomes derived from negative control transfected IESCs and the standard IESC derived exosomes. While no miR-182-5p silencing experiments were undertaken, Lai et al.’s (2020) findings suggest that a miR-182-5p/FOXO3 pathway is involved in gentamicin-induced ototoxicity.

### miRNA induced SIRTI reduction a common pathway in oxidative stress and diabetes mediated hair cell loss in ARHL

Xiong et al. (2015, 2019) studied the effects of miR-34a/SIRT1/p53 expression and hair cell death in ARHL. The authors studied the effects of oxidative stress on C57BL/6 mice and cultured HEI-OC1 cells. Auditory brainstem response thresholds increased at all tested frequencies with age, related to cochlear inner and outer hair cell loss. Overexpression of miR-34a inhibited SIRT1 causing increased p53 acetylation and hair cell apoptosis, thus causing hearing loss. Similarly, Xue et al. (2016, 2022) studied the effects of miR-29b on SIRT1/PGC-1α expression in C57BL/6 mice and HEI-OC1 cells in relation to AHL pathogenesis. The authors noted greater mitochondrial dysfunction in the cochleae of aged C75BL/6 mice and decreased hair cell count secondary to hair cell apoptosis. They identified that SIRT1 and PGC-1α expression was decreased in the cochlea of aged mice compared to young mice. Furthermore, miR-29b mimic transfection of HEI-OC1 cells suppressed SIRT1/PGC-1α levels whilst transfection with a miR-29b inhibitor upregulated the expression of SIRT1/PGC-1α. Therefore, the authors established that miR-29b overexpression leads to hair cell loss and mitochondrial dysfunction through the downregulation of SIRT1/PGC-1α signaling with aging.

Hao et al. (2019) investigated the association between a single nucleotide polymorphism of the myocardial ischemia associated transcript (MIAT) which is a long noncoding RNA in ARHL affected humans. The relationship between MIAT, SIRT1, and miR-29b in neuroblastoma cell lines was studied in young and aged C57BL/6 mice. They corroborated Xue et al.’s (2016) finding that PGC-1α/SIRT1 was decreased in aged mice and the frequency of cochlear mitochondrial dysfunction increased. They identified that an allelic polymorphism of MIAT increased the risk of ARHL in humans. Furthermore, MIAT modulated the effect of miR-29b on SIRT1 supporting a possible MIAT/miR-29b/PGC-1α/SIRT1signaling pathway in hair cell apoptosis and mitochondrial dysfunction in ARHL.

Homozygous leptin receptor deficient mice (db/db) that are known to be a good model of human type 2 diabetes (Burke et al., 2017) were compared to heterozygous controls by Lin et al. (2017). The db/db mice demonstrated cochlea hair cell loss, hearing impairment, elevated miR-34a, elevated mRNA levels of Hypoxia inducible factor (HIF), and reduced SIRT1 mRNA. miR-34a mimics had an identical effect on HEI-OC1 cells cultured under high glucose conditions, i.e., SIRT1 decreased and this was associated with increased HIF levels and HEI-OC1 cellular apoptosis. These findings provide further evidence for miRNAs exerting a deleterious effect on hearing via suppression of SIRT1 in association with a hypoxia/ischemia related co-factor.

### Potential novel treatments targeting cochlea hair cell depleting oxidative stress

Xiong et al. (2019) discovered that PGC-1α/NRF1/NRF2/TFAM, mitochondrial biogenesis-related proteins, were upregulated in aged C57BL/6 mice suggesting increased mitochondrial biogenesis in the aging cochlea. PINK1/Parkin signaling, characteristic of mitophagy, was also found to be increased. These changes were associated with an increase in mitochondrial mass but reduction in mitochondrial function, suggesting an accumulation of dysfunctional mitochondria with age. The authors concluded that resveratrol, a SIRT1 activator, counteracted the effects of cochlea aging by increasing mitophagy, attenuating increased mitochondrial biogenesis, and lead to improved mitochondrial function. miR-34 a deficient knock down mice were also found to exhibit reduced cochlear outer hair cells with age though less so than SIRT1 transgenic mice. Xiong et al.’s (2019) findings suggest that novel pharmaco therapies may have a role in slowing ARHL progression.

### Evidence from clinical practice and other sources relevant to hypoxamiRs in SNHL

The abrupt onset of sudden SNHL and the higher occurrence in metabolic syndrome is similar to acute myocardial infarction and stroke. This supports the ischemic theory of etiology in SSNHL. Autoimmune SNHL also typically presents with sudden hearing deterioration but differs from SSNHL because the episodes are recurrent and there is accompanying evidence of autoimmune disease. ARHL and NIHL the more predominant forms of SNHL occur gradually over years making it difficult to separate out any effect of hypoxic stress-related miRNAs from other causative factors. Therefore, studies of SSNHL and autoimmune SNHL are more likely to reveal information on hypoxia and hypoxic stress miRNAs in SNHL.

miR-132 and -195 were among eight differentially expressed miRNAs (DEMs) in the serum of SSNHL patients (Nunez et al., 2020). They have not so far been classified as hypoxamiRs however bioinformatic analysis reveals that they target RAF1 (proto-oncogene, serine/threonine kinase) which initiates the MAPK pathway. The MAPK signaling pathway, HIF, and VEGF are targets of identified hypoxamiRs (Gupta et al., 2018) supporting a hypoxic causal link in SSNHL. A JNK inhibitor (D-JNKI-1), a synthetic peptide was the active intervention agent in a phase 3 SSNHL clinical trial (ClinicalTrials.gov Identifier: NCT02561091). JNK1 is a member of the MAPK family, and while the outcome of the trial is not publicly available it means that therapeutic interventions targeting the MAPK pathway are feasible. miR-34a a hypoxamiR identified in this review as a SIRT1 inhibitor (Xiong et al., 2015, 2019) was differentially expressed in SSNHL patients’ plasma. Li et al. (2017) also predicted that MAPK pathway genes are likely targets of the 24 DEMS they identified in SSNHL patients. Furthermore, experimental induction of SSNHL by infusing lipopolysaccharides into the cochlea of guinea pigs reduced cochlear expression of apoptosis-inhibition genes Bcl-2 and Bcl-xl. Bcl-xl is linked by bioinformatic analysis to miR-204-5p an established hypoxamiR (Xie et al., 2021). Thus, there is complementary evidence from several sources suggesting that hypoxamiRs acting upon MAPK signaling pathways are important in the pathogenesis of SSNHL.

MAPK and vascular endothelial growth factor (VEGF) also play an important role in the innate immune system working through toll-like receptor (TLR) and interleukin-1β (IL-1β) receptor. MiRs -146 and -155 both target the common TL/IL-1 adaptor molecule myeloid differentiation primary response protein 88 (MYD88) as well as individually targeting TL and IL receptors and so suppressing the immune response. These miRs in turn are targeted by downstream MYD88 responsive molecules suggesting a miRNA/IL-1β/TL feedback mechanism fine tuning the innate immune response (Virtue et al., 2012). While the mechanisms leading to auto-immune SNHL are poorly understood. Pathak et al. (2011) found that auto-immune SNHL non-steroid responsive patients have higher levels of IL-1β and matrix metalloproteinase-9 (mmP-9) than responders. The higher levels of IL-1β and mmP-9 were reversed by an IL receptor agonist. The cause for IL-1β dysregulation is unknown but given the involvement of miRNAs -146, and -155 in an IL-1β feedback loop they are certainly promising candidates for further study.

## Limitations

Many included studies except Wang et al.’s (2010) study investigated the role of single selected miRNAs predominantly miR-34a and miR-29b in different types of acquired SNHL using animal models and/or *in vitro* cell models, thus precluding meta-analysis. No studies were identified that investigated the role of hypoxamiRs specifically in humans based on this review’s inclusion criteria reflecting a lack of human subject studies in this area.

## Author contributions

Review idea conception: DN and SS. Drafting the protocol: SS, PW, and DN. Study selection: SS, MN, and DN. Extracting data from studies: SS, MN, and PW. Synthesis and drafting the final review: SS, MN, PW, and DN. All authors contributed to the article and approved the submitted version.

## Funding

This review study is funded by a University of British Columbia, Faculty of Medicine, Summer Student Research Program (SSRP) award to SS, Pacific Otolaryngology Foundation, Rotary Club of Vancouver Hearing Foundation, and Vancouver Coastal Research Institute grants to DN.
